# Prevalence of frailty and its association with oral hypofunction in older adults: a gender perspective

**DOI:** 10.1186/s12903-023-02824-3

**Published:** 2023-03-10

**Authors:** Karla Cruz-Moreira, Ludwig Alvarez-Cordova, Carla González-Palacios Torres, Peter Chedraui, José Jouvin, José Juan Jiménez-Moleón, Rocío Barrios-Rodríguez

**Affiliations:** 1 Specialist in Oral Medicine, Dentistry degree, Catholic University of Santiago de Guayaquil, Guayaquil, Ecuador; 2 Instituto de Investigación e Innovación en Salud Integral, Catholic University of Santiago de Guayaquil, Guayaquil, Ecuador; 3 Master in Clinical Nutrition, Nutrition and Dietetics degree, Catholic University of Santiago de Guayaquil, Guayaquil, Ecuador; 4 Departamento de Medicina Preventiva y Salud Pública, Universidad de Granada, Granada, Spain; 5 Medical degree, PhD, Catholic University of Santiago de Guayaquil, Guayaquil, Ecuador; 6 Master in Health Services Management. Medical degree, Catholic University of Santiago de Guayaquil, Guayaquil, Ecuador; 7 Instituto de Investigación Biosanitaria ibs.GRANADA, Granada, Spain; 8 Centro de Investigación Biomédica en Red de Epidemiología y Salud Pública, Instituto de Salud Carlos III, Madrid, Spain

**Keywords:** Frailty, Oral hypofunction, Institutionalized elderly

## Abstract

**Background:**

Previous studies have indicated an association between oral hypofunction and frailty in community-dwelling older adults. However, this issue has not been evaluated in institutionalized older patients. We aimed to determine the prevalence of physical frailty in this particularly vulnerable group and evaluate its association with oral hypofunction, analyzing possible differences by gender.

**Methods:**

This cross-sectional study was conducted in private and public care homes in Guayaquil (Ecuador) from January 2018 until December 2019. Participants were classified as robust, pre-frail, and frail according to the Fried's frailty phenotype. Oral hypofunction was defined as the presence of at least three positive items in the following list: poor oral hygiene, oral dryness, reduced occlusal force, decreased masticatory function, and deterioration of swallowing function. The relationships between frailty and oral hypofunction were analyzed using logistic regression models for the whole sample and stratified by gender. Statistical analyses were performed using STATA 15.0 software (Stata Corp. LP, College Station, TX, USA).

**Results:**

Among the 589 participants analyzed (65% women), the median age was 72 years (interquartile range: 66–82). Pre-frailty and frailty were presented in 66.7% and 28.9% of them respectively. Weakness was the most frequent item (84.6%). There was a significant relationship between frailty and oral hypofunction in women. In the overall sample, the frequency of frailty was 2.06 times higher (95% CI 1.30–3.29) in patients with oral hypofunction, and this association was maintained in women (ORa: 2.18; 95% CI 1.21–3.94). Reduced occlusal force and decreased swallowing function were items significantly associated with the presence of frailty (ORa: 1.95; 95% CI 1.18–3.22 and ORa: 2.11; 95% CI 1.39–3.19, respectively).

**Conclusion:**

The prevalence of frailty and pre-frailty was high among institutionalized older people and was associated with the presence of hypofunction, especially in women. Decreased swallowing function was the most strongly item associated with frailty.

## Background

The world population older than 65 years is growing more rapidly than the population younger than that age. It is expected that the number of people in this age range will increase between 2022 and 2050, reaching 1.6 billion and more than twice the number of children under age 5 [1].

Aging is accompanied by changes in physical and psychological functions, with frailty as one of the most important concerns regarding the aging population [2]. Frailty is a multidimensional syndrome characterized by reduced resistance and physiological reserve and increased vulnerability to endogenous and exogenous stressors [3]. This syndrome is related to increased hospitalization, mortality risk, and long-term care use, and its associated economic cost is expected to increase substantially over the time [4–7]. Although there is a lack of consensus around a single operational definition of frailty, three operational definitions have been proposed [8]. One is predominantly based on the physical condition. For example, the physical/biological construct of Fried and colleagues that considers five physical items for a frailty phenotype measurement [9]. A second comprises a deficit accumulation model, which considers symptoms, signs, functional impairments, and laboratory abnormalities. The last model is based on a combination of physical and psychosocial aspects and is the most widely used operational definition of frailty. Several tools exist to evaluate frailty using these different approaches; however, none is considered the gold standard [10, 11]. Nonetheless, the phenotypic approach developed by Fried et al. is one of the most widely used [12, 13].

There are important differences in the prevalence of frailty in older people around the world (from 3.9 to 51.4%), and it is higher in upper-middle-income countries and in women [14, 15]. For example, it has been reported that the prevalence of frailty and pre-frailty in Ecuador reaches 44% [16]. This data is even higher in institutionalized older people [17]. Moreover, it has been suggested that there are clear gender differences for frailty, with frailty being present more often among females than males at all ages [18]. Identifying frailty and modifiable factors associated with it are essential to develop and implement effective preventive interventions [2, 3], and the consideration of gender is relevant to this issue [18].

In this sense, an association has been suggested between preventable alterations of the oral cavity and frailty in communities-dwelling older adults [19, 20]. In 2016, the Japanese Society of Gerontology proposed a new clinical concept, oral hypofunction, which includes multiple oral functions [21]. It is not a structural condition, but a functional, physiological condition comprising three or more of the following indicators: poor oral hygiene, oral dryness, reduced occlusal force, decreased tongue-lip motor function, decreased tongue pressure, decreased masticatory function, and deterioration of swallowing function. The few previous studies analysing the association between oral hypofunction and frailty have shown a higher frequency of the frailty among community-dwelling older adults with oral hypofunction or/and with deterioration of some of its components [22–24].

Given the special interest in preventing frailty in older people due to its negative consequences, the wide variations in the prevalence of this syndrome around the world, the described association between oral hypofunction and frailty in community-dwelling older adults, and the absence of studies analyzing this issue in an especially vulnerable group, institutionalized older people, this study aimed: (1) to determine the prevalence of frailty in institutionalized older people and (2) to evaluate its association with oral hypofunction, analyzing possible differences by gender.

## Methods

### Study design and participants

The data collection for this cross-sectional study was carried out from January 2018 until December 2019 in Guayaquil, Ecuador. Oral examinations were conducted by three dentists. In addition, three dietitian nutritionists were involved for some of the frailty measurement items, such as exhaustion, low physical activity, and slowness (see their description below). The team members participated in calibration sessions before the start of the study. Ten older adults were examined for the calculation of inter-examiner reliability. Interclass correlation coefficients ranged from 0.97 to 0.99 among both the dentists and dietitian nutritionists.

The study participants were institutionalized people in the 10 private and public care homes of Guayaquil. The care homes were: *Hogar Luis Plaza Dañin, Asilo Hogar San José, Centro Gerontológico Vida Plena, Asilo Corazón de Jesús/Junta de Beneficencia de Guayaquil, Hogar la Esperanza #2, Centro gerontológico Sofía Ratinoff, Centro gerontológico Municipal Iglesia elevación, Centro gerontológico Municipal Orquídeas, Centro gerontológico Municipal Dr. Arsenio de la Torre Marcillo* and *Club del adulto mayor*. The care home *La Casa del Hombre Doliente* was not selected because it is dedicated to older adults in the terminal phase of an illness.

Participants were included after they, or their surrogate, agreed to participate in the study and signed the informed consent. The Ethics Committee of Hospital Clínica Kennedy (HCK-CEISH-19-0036) approved this study.

The inclusion criteria were: (1) permanent or temporary institutionalized older person in private or public care homes in Guayaquil city, (2) participants aged ≥ 65, 3) older adults without mild or severe cognitive impairment as shown by the Mini-Mental State Examination (MMSE) (score > 23/30) [25] due to possible barriers in their collaboration in data collection. The exclusion criteria were: (1) residents with a medical history of stroke, ischemic heart disease, Parkinson´s disease, or Alzheimer disease because these functional pathologies occupy between 52 and 84% of the causes of the dysphagia [26], and (2) older adults unable to walk due to a health problem because they cannot perform the speed test to evaluate slowness.

### Frailty evaluation

The criteria and the methods proposed by Fried et al. were used for the evaluation of frailty [9]. The frailty phenotype considers five components: exhaustion, low physical activity, slowness, weakness, and weight loss. Participants with three or more components were considered frail, and those with one or two components were considered pre-frail. Participants without any component were considered robust. Detailed criteria for each phenotype are defined below.

1. Exhaustion. Two items in the Centre for Epidemiologic Studies Depression Scale were used to evaluate exhaustion: “I felt that everything I did was an effort last week”, and “I couldn´t get going last week”. Presence of exhaustion was considered if the participant answered “frequently” or “always” in either of the two questions.
2. Low physical activity. The participants were asked about their physical activities using the Minnesota Leisure Time Activity Questionnaire (MLTAQ). A weighted score of kilocalories (kcal) expended per week was calculated. Men with < 383 kcal and women with < 270 kcal were considered positive for this component.
3. Slowness: A stopwatch was used to measure the time taken to walk 4.6 m in the speed test. The positive criterion of frailty in men was (height-time) ≤ 173 cm ≥ 7 s or > 173 cm ≥ 6 s; in women, it was considered positive if (height-time) ≤ 159 cm ≥ 7 s or > 159 cm ≥ 6 s.
4. Weakness: We used a dynamometer (Jamar TM Hydraulic Hand Dynamometer 5030 J1) to measure grip strength in the dominant hand. Strength was adjusted for sex and body mass index (BMI) and was recorded in kilograms using the criteria established by Fried et al. [9].
5. Weight loss: Their weight during the previous year was found in the medical records of the care home, and the current weight was measured. A weight loss ≥ 10 pounds (4.5 kg), unintentional in the last year compared with the prior year was considered positive for this item.

### Oral hypofunction

Seven conditions are considered to evaluate oral hypofunction: poor oral hygiene, oral dryness, reduced occlusal force, decreased tongue-lip motor function, decreased tongue pressure, decreased masticatory function, and deterioration of swallowing function. Nevertheless, it is also possible to evaluate it with five of the seven items [21]. Thus, in this study, we did not consider decreased tongue-lip motor function or decreased tongue pressure due to the difficulty of assessing these. Each item was assigned 0 if it was negative or 1 point if it was positive. If the final score, total sum of the items, was 3 points or higher, the person was considered to have oral hypofunction.

The Oral Hygiene Index (OHI) was used to assess for poor oral hygiene [27]. For this method, the debris and the calculus of four surfaces of the teeth were examined (labial/buccal, palatal/lingual, mesial, and distal). The codes used were from 0 (no debris or no calculus) to 3. The mean values were obtained and summed. Thus, as described previously [28], the rating scale was: 0.0–1.2: good oral hygiene, 1.3–3.0: average oral hygiene, and 3.1–6.0: poor oral hygiene. As previous studies evaluating the associations between oral hypofunction and frailty used the 50% as a cut-off point to consider poor oral hygiene [23, 24], average and poor hygiene were considered positive for oral hypofunction in this study.

The sialometry was used to evaluate oral dryness, obtaining the unstimulated whole saliva using the draining method. Patients were instructed to refrain from eating, drinking, brushing teeth, using mouthwash, or smoking for 60 min prior to the evaluation. All assessments were made between 8:00 am and 10:00 am. This item was considered positive in oral hypofunction if the salivary flow was less than 0.25 ml/min [29–31].

Oral hypofunction diagnostic criteria indicate that occlusal force can be assessed using the number of remaining teeth. Thus, the diagnosis of reduced occlusal force was made when the number of natural teeth is less than 20, excluding remaining roots and teeth with considerable mobility (mobility 3) [21, 24].

Decreased masticatory function was determined by using 2-colored chewing gum (Hubba-Bubba tape gum in the flavors Sour Berry, blue, and Fancy Fruit, pink). The gum was masticated for a total of 20 strokes, and then was flattened between 2 transparent glass slides, creating a 1-mm-thick bolus that was subsequently analyzed. All the masticated boluses were analyzed using the Chewing Performance Calculator (CPC) https://studio.chewing.app/. A detailed description of the method is available here [32, 33]. The mixing ability was recorded as a percentage. A decreased masticatory function was considered when the percentage was lower than the 75^th^ percentile of the sample (75.5). This value was close to the value from which it is considered good chewing [32].

The deterioration of swallowing function was assessed using the questionnaire for swallowing screening (a 10 item Eating Assessment Tool) [22]. A total score of ≥ 3 indicated reduced swallowing function.

### Other variables

Data about sociodemographic aspects (sex, age, level of studies, marital status), smoking, alcohol consumption (any level of daily tobacco— ≥ 1 cig/day—and weekly alcohol— ≥ 1 drink/week—was considered to classify participants as smokers and alcohol consumers, respectively), and current chronic diseases (hypertension, diabetes mellitus, heart disease, osteoarticular disease, osteoporosis, etc.) were collected using a standardized questionnaire.

### Data management and statistics

The results are expressed as means and standard deviations. A Chi-square test was performed to evaluate the associations between sociodemographic characteristics and the presence of frailty. The potential modifying effect of sex on the associations of hypofunction oral and frailty was tested through the interaction by entering the product term in each model. Despite these results, we performed sex-stratified analysis because sex differences in the burden of frailty and a higher prevalence of oral hypofunction in women have been reported previously [18, 34, 35]. Logistic regression models for overall and stratified by sex were performed. Two logistic models were run: (i) Model 1 presents crude odds ratios (OR) and 95% confidence intervals (95% CI), (ii) Model 2, adjusted for age, sex (just in the overall models), level of studies, marital status, presence of chronic diseases, smoking and alcohol consumption. The second model was constructed based on prior knowledge, adjusting by those variables that the scientific literature has related to frailty and oral hypofunction. Statistical analyses were performed using STATA 15.0 software (Stata Corp. LP, College Station, TX, USA).

## Results

**Fig. 1:**
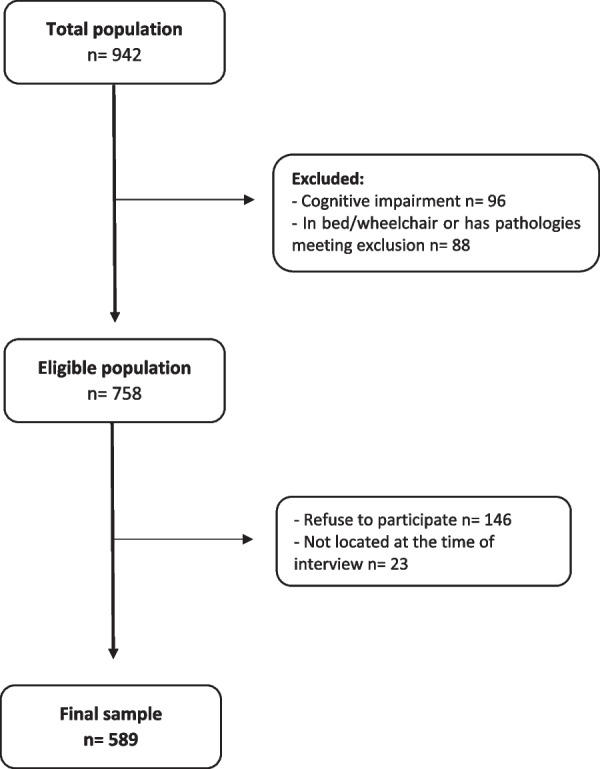
Flowchart of participants

Of the 942 institutionalized elderly people on the day of data collection, 184 were excluded (96 had cognitive impairment, and 88 were bedbound or in a wheelchair for different pathologies). The eligible population was 758, of which 146 refused to participate (19.3%), and 23 (3.0%) were not located at the time of the interview, obtaining a final sample of 589 (Fig. 1). The main characteristics of the sample are shown in Table 1. In the total sample, the median age was 72 years (interquartile range: 66–82). Only 16.6% of the sample had a higher education level, and 28% were married. With respect to habits, the majority did not smoke (77.2%) or drink alcohol (67.9%). About half of the participants had a chronic disease (49.8%). The presence of weakness was the most frequent frailty item (84.6%), followed by slowness (77.4%). Regarding gender, more than half of the participants were women (65%). Men were older, more likely to be married, to be a smoker, and to drink alcohol than women. Chronic diseases were present more frequently in women. Regarding components of frailty, women had more presence of low physical activity but less presence of slowness and weakness.

**Table 1:** Sociodemographic characteristics, habits, and prevalence of frailty in the total sample and by gender

	Overall (n = 589)	Men (n = 206)	Women (n = 383)	*p*-value
	n (%)	n (%)	n (%)	
Age (years), median (IQR)^a^	72 (66–82)	74 (67–84)	71 (65–81)	0.028^b^
65–69	229 (38.9)	73 (35.5)	156 (40.7)	0.239^c^
70–74	99 (16.8)	32 (15.5)	67 (17.4)	
75–100	261 (44.3)	101 (49.0)	160 (41.8)	
Education level				0.453^c^
Incomplete/primary education	315 (53.5)	104 (50.5)	211 (55.1)	
Secondary education	176 (29.9)	63 (30.6)	113 (29.5)	
Higher education	98 (16.6)	39 (18.9)	59 (15.4)	
Marital status				0.002^c^
Married	165 (28.0)	73 (35.4)	92 (24.0)	
Single	191 (32.4)	69 (33.5)	122 (31.9)	
Divorced/separated/widowed	233 (29.6)	64 (31.1)	169 (44.1)	
Current smoking				< 0.001^c^
No	455 (77.2)	122 (59.2)	333 (87.0)	
Yes	134 (22.8)	84 (40.8)	50 (13.0)	
Current alcohol drinking				< 0.001^c^
No	400 (67.9)	90 (43.7)	310 (80.9)	
Yes	189 (32.1)	116 (56.3)	73 (19.1)	
Presence of chronic diseases^d^				< 0.001^c^
No	296 (50.2)	127 (61.6)	169 (44.1)	
Yes	293 (49.8)	79 (38.4)	214 (55.9)	
Presence of components of frailty				
Exhaustion	102 (17.3)	33 (16.0)	69 (18.0)	0.323^c^
Low physical activity	12 (2.0)	8 (3.9)	4 (1.0)	0.020^c^
Slowness	456 (77.4)	146 (70.9)	310 (80.9)	0.005^c^
Weakness	498 (84.6)	183 (88.8)	315 (82.3)	0.035^c^
Weight loss	143 (24.3)	48 (23.3)	95 (24.8)	0.685^c^

^a^IQR: interquartile range; ^b^Mann–Whitney U test; ^c^Chi-square test; ^d^Hypertension, diabetes mellitus, heart disease, osteoarticular disease, osteoporosis, mainly

Regarding the presence of frailty and its association with sociodemographic characteristics, just 4.4% were robust; the rest of participants presented some grade of frailty (Table 2). Age and the presence of chronic diseases were the only variables significantly associated with frailty. Robust women were younger than those with presence of prefailty or frailty (Table 3). Frail women were more frequently divorced, separated, or widowed and had chronic diseases with more frequency.

**Table 2:** Associations between sociodemographic characteristics and presence of frailty in institutionalized older adults (n = 589)

	Robust n (%)	Pre-frail n (%)	Frail n (%)	*p*-value^a^
All	26 (4.4)	393 (66.7)	170 (28.9)	
Gender				0.171
Woman	14 (3.7)	265 (69.2)	104 (27.1)	
Men	12 (5.8)	128 (62.1)	66 (32.1)	
Age (years)				0.032
65–69	15 (57.7)	159 (40.4)	55 (33.3)	
70–74	1 (3.8)	71 (18.1)	27 (15.9)	
75–100	10 (38.5)	163 (41.5)	88 (51.8)	
Education level				0.489
Incomplete/primary education	12 (3.8)	203 (64.4)	100 (31.7)	
Secondary education	10 (5.7)	121 (68.8)	45 (25.5)	
Higher education	4 (4.1)	69 (70.4)	25 (25.5)	
Marital status				0.102
Married	8 (30.8)	118 (30.0)	39 (23.0)	
Single	9 (34.6)	133 (33.8)	49 (28.8)	
Divorced/separated/widowed	9 (34.6)	142 (36.1)	82 (48.2)	
Current smoking habit				0.503
No	20 (4.4)	309 (67.9)	126 (27.7)	
Yes	6 (4.5)	84 (62.7)	44 (32.8)	
Current drinking habit				0.140
No	15 (3.8)	277 (69.2)	108 (27.0)	
Yes	11 (5.8)	116 (61.4)	62 (32.8)	
Presence of chronic diseases^b^				0.047
No	19 (6.4)	197 (66.6)	80 (27.0)	
Yes	7 (2.4)	196 (66.9)	90 (30.7)	

^a^Chi-square test; ^b^Hypertension, diabetes mellitus, heart disease, osteoarticular disease, osteoporosis, mainly

**Table 3:** Associations between sociodemographic characteristics and presence of frailty by gender

	Men				Women			
	Robust n (%)	Pre-frail n (%)	Frail n (%)	*p*-value^a^	Robust n (%)	Pre-frail n (%)	Frail n (%)	*p*-value^a^
Age (years)				0.189				0.023
65–74	4 (33.3)	71 (55.5)	30 (45.5)		12 (85.7)	159 (60.0)	52 (50.0)	
75–100	8 (66.7)	57 (44.5)	36 (54.5)		2 (14.3)	106 (40.0)	52 (50.0)	
Education level				0.945				0.479
Incomplete/primary education	6 (50.0)	62 (48.5)	36 (54.5)		6 (42.9)	141 (53.2)	64 (61.5)	
Secondary studies	4 (33.3)	41 (32.0)	18 (27.3)		6 (42.9)	80 (30.2)	27 (26.0)	
Higher studies	2 (16.7)	25 (19.5)	12 (18.2)		2 (14.1)	44 (16.6)	13 (12.5)	
Marital status				0.944				0.044
Married	4 (33.3)	47 (36.7)	22 (33.3)		4 (28.6)	71 (26.8)	17 (16.4)	
Single	4 (33.3)	44 (34.4)	21 (31.9)		5 (35.7)	89 (33.6)	28 (26.9)	
Divorced/separated/widowed	4 (33.3)	37 (28.9)	23 (34.8)		5 (35.7)	105 (39.6)	59 (56.7)	
Current smoking habit				0.684				0.169
No	8 (66.7)	73 (57.0)	41 (62.1)		12 (85.7)	236 (89.1)	85 (81.7)	
Yes	4 (33.3)	55 (43.0)	25 (37.9)		2 (14.3)	29 (10.9)	19 (18.3)	
Current drinking habit				0.951				0.266
No	5 (41.7)	57 (44.5)	28 (42.4)		10 (71.4)	220 (83.0)	80 (76.9)	
Yes	7 (58.3)	71 (55.5)	38 (57.6)		4 (28.6)	45 (17.0)	24 (23.1)	
Presence of chronic diseases^b^				0.924				0.017
No	8 (66.7)	79 (61.7)	40 (60.6)		11 (78.6)	118 (44.5)	40 (38.5)	
Yes	4 (33.3)	49 (38.3)	26 (39.4)		3 (21.4)	147 (55.5)	64 (61.5)	

^a^Chi-square test; ^b^Hypertension, diabetes mellitus, heart disease, osteoarticular disease, osteoporosis

Oral hypofunction data for the sample is presented in Table 4. 71% of the institutionalized older people had oral hypofunction. The components with a higher frequency of appearance were poor oral hygiene (81.5%), reduced occlusal force (76.6%), and decreased masticatory function (75.0%).

**Table 4:** Description of oral hypofunction and its components in overall institutionalized older adults (n = 589)

	n (%)
Oral hypofunction	
No	171 (29.0)
Yes	418 (71.0)
Poor oral hygiene	
No	109 (18.5)
Yes	480 (81.5)
Oral dryness	
No	309 (52.5)
Yes	280 (47.5)
Reduced occlusal force	
No	138 (23.4)
Yes	451 (76.6)
Decreased masticatory function	
No	147 (25.0)
Yes	442 (75.0)
Decreased swallowing function	
No	452 (76.7)
Yes	137 (23.3)

Table 5 shows the associations between oral hypofunction and its components with frailty, and also stratified by sex. The frequency of frailty was 2.06 times higher (95% CI 1.30–3.29) in patients with oral hypofunction in the adjusted model (*p*-value of interaction for sex: 0.583); however, this significant association was only maintained in women (ORa: 2.18; 95% CI 1.21–3.94). The presence of frailty was significantly associated with reduced occlusal force (ORa: 1.95; 95% CI 1.18–3.22) and decreased swallowing function (ORa: 2.11; 95% CI 1.39–3.19) (*p*-value of interaction for sex: 0.850 and 0.089, respectively). The magnitude of the associations was similar among women. Decreased masticatory function had an inverse relationship with the presence of the frailty in men (ORa: 0.48; 95% CI 0.25–0.96).

**Table 5:** Association between oral hypofunction with the presence of frailty in the overall sample and stratified by gender

	Overall n = 589		Men n = 206		Women n = 383	
	Model 1^a^	Model 2^b^	Model 1^a^	Model 2^b^	Model 1^a^	Model 2^b^
	OR (95% CI)	OR (95% CI)	OR (95% CI)	OR (95% CI)	OR (95% CI)	OR (95% CI)
Oral hypofunction						
No	1.00	1.00	1.00	1.00	1.00	1.00
Yes	**2.37 (1.52–3.69)**	**2.06 (1.30–3.29)**	2.00 (0.95–4.21)	1.86 (0.84–4.08)	**2.54 (1.46–4.42)**	**2.18 (1.21–3.94)**
Poor oral hygiene						
No	1.00	1.00	1.00	1.00	1.00	1.00
Yes	1.08 (0.68–1.72)	0.94 (0.58–1.53)	1.16 (0.46–2.96)	1.13 (0.43–2.99)	1.01 (0.59–1.73)	0.84 (0.47–1.49)
Oral dryness						
No	1.00	1.00	1.00	1.00	1.00	1.00
Yes	1.40 (0.98–2.00)	1.29 (0.89–1.88)	1.18 (0.66–2.13)	1.09 (0.59–2.03)	1.57 (1.00–2.47)	1.44 (0.89–2.32)
Occlusal force						
Not reduced	1.00	1.00	1.00	1.00	1.00	1.00
Reduced	**2.14 (1.33–3.45)**	**1.95 (1.18–3.22)**	**2.28 (1.03–5.05)**	2.17 (0.94–5.00)	**2.06 (1.14–3.73)**	1.85 (0.98–3.49)
Masticatory function						
Not decreased	1.00	1.00	1.00	1.00	1.00	1.00
Decreased	0.73 (0.48–1.09)	0.67 (0.45–1.03)	**0.49 (0.25–0.96)**	**0.48 (0.25–0.96)**	0.88 (0.53–1.46)	0.80 (0.47–1.35)
Swallowing function						
Not decreased	1.00	1.00	1.00	1.00	1.00	1.00
Decreased	**2.32 (1.56–3.47)**	**2.11 (1.39–3.19)**	1.52 (0.82–2.83)	1.43 (0.76–2.69)	**3.08 (1.81–5.25)**	**2.83 (1.63–4.91)**

^a^Unadjusted logistic regression model with presence of frailty as dependent variable; ^b^Logistic regression model with presence of frailty as dependent variable and adjusted for age, sex (just in overall models), level of studies, marital status, presence of chronic diseases, smoking and alcohol consumption. Bold font indicates statistical significance

## Discussion

To our knowledge, this is the first study evaluating the prevalence of frailty in institutionalized older people and its association with oral hypofunction. The wide variation in the prevalence of frailty and pre-frailty makes its evaluation necessary in specific contexts. The findings of this study showed that the prevalence of pre-frailty and frailty was high, with weakness being the component most frequently affected. The presence of frailty was associated with oral hypofunction, especially with decreased swallowing function, but the significant association was only maintained in women.

The prevalence of frailty reported in literature ranges widely, depending on study design, population, and setting. However, the number of studies focused on the institutionalized population is scarce. A 2015 meta-analysis found large heterogeneity in the prevalence of frailty in nursing homes, with pooled estimates of 52.3% (95% CI 37.9–66.5%) and 40.2% (95% CI 28.9–52.1%) for frailty and pre-frailty, respectively [17]. More recent studies in residential aged care facilities have found an even higher prevalence of frailty [36–38], which is higher than that described in this study. These discrepancies may be partially due to the difference between the mean ages of the participants included in this study (74.7 years) and in the previous one (more than 80 years), as it is well-established that frailty is an age-related clinical condition. Nevertheless, it is noteworthy that only 4.4% of our participants were robust, and more than 65% of our participants had pre-frailty. Pre-frailty is a condition that predisposes, and usually precedes, the frailty state, so its identification may present an opportunity to introduce effective prevention strategies [39].

Regarding oral hypofunction, we found this to be impaired in our participants than that previously described by other studies in non-institutionalized people (ranging from 35.9 to 63%) [23, 34, 40–42]. Variations in the educational level, presence of chronic diseases or habits among samples may explain this finding when they are associated factors with oral health status [43]. However, these data were not described in the majority of the referenced studies compared. Another possible explanation for this data difference could be that our population is institutionalized older adults, and therefore they could be more dependent than those in other studies. In this sense, it has also been reported that caregivers' lack of time, knowledge, and/or training can be barriers to providing oral care [44]. Although the causes of reduction in oral hygiene are multifactorial, this reason is also compatible with poor oral hygiene being the most impaired item, similar to that reported by previous studies (prevalence of 72.8% and 92.8%) [22, 24]. Even though other authors have reported that poor oral hygiene was the least common component, [34, 40] it is still essential to establish effective strategies to overcome barriers, such as oral health education programs, and to increase facilitators in providing oral care in institutionalized older adults.

Interestingly, when we stratified the statistical analyses by gender, the significant association between oral hypofunction and frailty was only significant in women and not in men. This gender difference has been previously described in the relationship between oral hypofunction and physical frailty [45]. Although the effect of gender on frailty remains poorly understood, the prevalence of frailty is consistently higher in women than in men [18, 35]. Likewise in our study, although it was not a statistically significant difference. Therefore, the association with oral hypofunction only in women could be relevant in prevention terms. Of note the decreased swallowing function was the strongest single item associated with frailty in women, even more than that of oral hypofunction as a whole. When swallowing dysfunction is a preventable process, it may be key in the intervention of frailty. Oral aspects such as the loss of teeth have been closely associated with reduced swallowing function [46, 47]; however this function might not improve with dentures [48]. In fact, in our sample, 74.2% of the women used a removable prosthesis. Therefore, these results along with the described contribution of swallowing function in other adverse health-related outcomes, such as hospitalization or mortality [49], highlight the relevance of evaluating this function and implementing appropriate dental intervention, especially in women.

Unexpectedly, we found an inverse relationship between decreased masticatory function and the presence of frailty in men. This association could be confounded by other factors or it could be a real association. For example, masticatory ability has been related to occlusal force and maximum tongue pressure, indicating that the large muscle mass in the oral cavity is indispensable for improving masticatory function [50]. In this sense, a sex difference in muscle strength has been previously suggested and could be behind the inverse association found [41].

This study presents some limitations. First, it is a cross-sectional study design where the participants were assessed only once. Therefore, it is not possible to conclude a causal association between oral hypofunction and frailty. However, a clinical cascade between oral hypofunction and physical frailty has been verified previously [45]. Second, frailty was evaluated with Fried´s Frailty Phenotype, which could be less feasible to include in the clinical practice because it requires measurement of grip strength, for example. Nevertheless, this method is one of the most widely used and has high validity and reliability [13]. Third, as previously mentioned, 5 of the 7 components of oral hypofunction were evaluated, which could have underestimated the prevalence. In addition, there is diversity in the evaluation of the items between the different studies that also may affect the prevalence values. Nevertheless, this was not the main objective of this study. Fourth, data collection was performed in 2018–2019, which may have had an impact on our results. This circumstance could have produced an underestimation of the results taking into account the expected increase in the prevalence of frailty over time due to population aging. Finally, although we have analyzed potential factors associated with frailty in institutionalized people, we cannot rule out the existence of other variables that influence on it.

## Conclusion

The prevalence of frailty and pre-frailty found among institutionalized older people was high and was associated with the presence of oral hypofunction, especially in women. Decreased swallowing function was the item most strongly associated with frailty. Longitudinal studies are needed to evaluate the contribution of oral function in frailty development and progression and further address sex-dependent changes in this relationship.

## Data Availability

The data that support the findings of this study are available from the corresponding author upon reasonable request.

## Abbreviations

MMSE: Mini-mental state examination
CESD-R: Centre for epidemiologic studies depression scale
MLTAQ: Minnesota leisure time activity questionnaire
KCAL: Kilocalories
BMI: Body mass index
OHI: Oral hygiene index
CPC: Chewing performance calculator
EAT-10: 10 item eating assessment tool
OR: Odds ratio
SD: Standard deviation
